# Human lung cancer-derived mesenchymal stem cells promote tumor growth and immunosuppression

**DOI:** 10.1186/s13062-024-00479-w

**Published:** 2024-05-16

**Authors:** Xiaoyan Gao, He Ren, Zhengrong Zhang, Shuai Cao, Bo Zhang, Qiang Sun, Gerry Melino, Hongyan Huang

**Affiliations:** 1 Department of Oncology, Beijing Shijitan Hospital of Capital Medical University, 10 Tieyi Road, Beijing, 100038 China; 2 Department of Experimental Medicine, TOR, University of Rome “Tor Vergata”, Rome, Italy; 3 Department of Orthopedics, Civil Aviation General Hospital, No.1 Gaojing Street, Chaoyang District, Beijing, 100123 China; 4 Laboratory of Cell Engineering, Institute of Biotechnology, Beijing, China; 5 DZNE German Center for Neurodegenerative Diseases, Bonn, Germany

**Keywords:** Mesenchymal stem cell, Non-small cell lung cancer, Immunophenotype, Tumorigenic, Multipotent differentiation, Natural killer cell, T cell

## Abstract

**Background:**

The presence of mesenchymal stem cells has been confirmed in some solid tumors where they serve as important components of the tumor microenvironment; however, their role in cancer has not been fully elucidated. The aim of this study was to investigate the functions of mesenchymal stem cells isolated from tumor tissues of patients with non-small cell lung cancer.

**Results:**

Human lung cancer-derived mesenchymal stem cells displayed the typical morphology and immunophenotype of mesenchymal stem cells; they were nontumorigenic and capable of undergoing multipotent differentiation. These isolated cells remarkably enhanced tumor growth when incorporated into systems alongside tumor cells in vivo. Importantly, in the presence of mesenchymal stem cells, the ability of peripheral blood mononuclear cell-derived natural killer and activated T cells to mediate tumor cell destruction was significantly compromised.

**Conclusion:**

Collectively, these data support the notion that human lung cancer-derived mesenchymal stem cells protect tumor cells from immune-mediated destruction by inhibiting the antitumor activities of natural killer and T cells.

## Background

Mesenchymal stem cells (MSCs) are fibroblast-like, multipotent progenitor cells that were initially discovered in bone marrow; however, over time, their presence has been confirmed in almost all tissue types, and these cells exhibit the potential for multidirectional differentiation (such as into bone, adipose, cartilage, and muscle cells) as well as the capacity for self-renewal [1–3]. MSCs can be recruited to the site of tissue injuries where they participate in processes associated with wound repair. Tumors are considered to be "wounds that never heal," and MSCs tend to migrate toward sites of inflammation and tumor microenvironments [3, 4]; therefore, many studies have recommended the use of MSCs as therapeutic vectors to target tumors [5–7]. However, the function of MSCs in cancer remains controversial, and it is essential to clarify their effects within the tumor microenvironment. Several studies have demonstrated that MSCs exert their antitumor effects through several mechanisms, including the inhibition of angiogenesis, the promotion of antitumor immune responses, and the induction of apoptosis in cancer cells [8–10]. In contrast, several other studies have shown that MSCs can *promote* tumor growth and metastasis by enhancing tumor cell proliferation, angiogenesis, and metastatic capacity, thereby inducing immunosuppression or inhibiting tumor cell apoptosis [11–17]. Possible explanations for the discrepancies between studies could be the variability of MSCs in terms of the tissue and microenvironment from which they were isolated or the fact that some studies have used murine rather than human MSCs. Notably, previous studies have mainly focused on MSC lines or healthy donor-derived MSCs isolated from bone marrow. Only recently has there been growing interest in the effects of tumor-derived MSCs on cancer progression. However, the interactions between tumor-associated MSCs and cancer cells remain obscure and require further investigation.

The aims of this study were to isolate human lung cancer-derived MSCs (hLC-MSCs) to characterize their phenotypes, assess their effects on tumor growth both in vivo and in vitro, and elucidate the mechanisms underlying their tumor-promoting effects.

## Materials and methods

### Dissociation of tumor-associated mesenchymal cells

Tumor tissue samples were obtained from two patients with non-small cell lung cancer (NSCLC) who had not received any treatment prior to undergoing surgical resection at Xuanwu Hospital. Written informed consent was obtained according to the guidelines of the Ethics Committee of Capital Medical University. Lung tumor tissues were minced and added to a solution containing a mixture of dispase and collagenase 1A (STEMCELL Technologies Inc, Vancouver, BC, Canada) for digestion. A cell strainer (70 μm; BD Biosciences, Bedford, MA) was used for single-cell isolation and the removal of adipose and other tissues.

### Cells and culture conditions

hLC-MSC cell expansion was performed as previously described by Liu et al. [18]. Briefly, isolated epithelial cells were co-cultivated with irradiated (3,000 rad) Swiss 3T3 fibroblasts (J2 strain) in F medium [3:1 (v/v) Ham’s Nutrient Mixture F-12: Dulbecco's Modified Eagle's Medium (Invitrogen), with 5% fetal bovine serum (FBS), 10 ng/mL epidermal growth factor (Invitrogen, Waltham, MA), 5 μg/mL insulin, 0.4 μg/mL hydrocortisone, 24 μg/mL adenine (Sigma-Aldrich, St. Louis, MO), and 8.4 ng/mL cholera toxin], to which 10 μmol/L Y-27632 (ROCK inhibitor, Tocris Bioscience, Bristol, UK) was added. All cells were cultured in a humidified atmosphere of 5% CO_2_ at a temperature of 37 °C and passaged at a ratio of 1:4 after reaching 80%–90% confluence.

Differential trypsinization was performed to separate feeder and epithelial cells during passaging. Briefly, feeder/epithelial co-cultures were rinsed with phosphate-buffered saline (PBS) and incubated with 0.05% trypsin solution at room temperature for 30 s to 1 min, with close monitoring under phase-contrast microscopy. When the feeder cells became rounded and began to detach from the substrate, the cultures were gently tapped to facilitate their detachment and subsequent removal by aspiration, while the epithelial cell colonies remained tightly adherent. The epithelial cells were again rinsed with PBS and trypsinized at 37 °C for 3–5 min. The cells were transferred to a solution of PBS containing 10% serum to neutralize the trypsin and subjected to centrifugation at 500×*g*. The cell pellets were subsequently resuspended in F medium for passaging. To minimize any potential changes in cellular behavior caused by prolonged culture times, cells at passages P3–P5 were used in this study.

Cell line MC38/CT-26/NIH3T3/A549/HepG2 and its derivatives were routinely maintained in Dulbecco’s Modified Eagle’s Medium (MACGENE Technology Ltd., Beijing, China) supplemented with 10% FBS (Kangyuan Biology, China), and 100 units/mL penicillin plus 100 µg/mL streptomycin (Invitrogen). NK92MI cell line was maintained in RPMI 1640 medium (MACGENE Technology Ltd.) supplemented with 12.5% FBS and 12.5% horse serum (Kangyuan Biology). T cells isolated from peripheral blood and activated were further cultured in RPMI 1640 medium supplemented with 10% FBS supplemented with 100 IU/mL human interleukin 2 (hIL-2). All cells were cultured in a humidified incubator with 5% CO2 at 37 °C.

### Constructs and stable cell lines

The *luciferase* gene was subcloned into a pQCXIP retroviral vector to generate a pQCXIP-luciferase-puro vector. pQCXIP and retroviral helper plasmids vesicular stomatitis virus G (VSV-G) and Gag-Pol-Rev were purchased from Addgene. All constructs were verified using DNA sequencing. More detailed information is provided below.

**Table:** 

Plasmid name	Construct method	Vector backbone	Cleavage site	DNA	Primer sequence (5' → 3')
pQCXIP-luciferase	Homologous recombination	pQCXIP-Puro A	XhoI	*luciferase*	AGCTTGGTACCGAGCTCgCAGTGCGTCAATCTGACAACTCG
			BamHI		TTCGGGCCCTCCTCGAGCGGTGTAATGCAGCTTCACGC

Stable cell lines were established via viral infection. Retroviruses were packaged into human embryonic kidney 293 T (HEK293T) cells using Lipofectamine 2000 reagent (Invitrogen), as previously described [19, 20]. For infection, 1 mL of viral supernatants mixed with 10 μg of Polybrene (Sigma) was added to the target cells in 6-well plates for a period of 12 h, after which the cells were fed with regular media. Virus-infected tumor cells were selected through a 7–14-day exposure to puromycin (2 μg/mL).

### Flow cytometric analysis

Cells were detached from the culture plate using TrypLE Express (Invitrogen) before being resuspended in PBS supplemented with 1% bovine serum albumin. The cells were incubated with fluorophore-conjugated antibodies targeting various cluster of differentiation (CD) and human leukocyte antigens (HLA) for 30 min in the dark at 4 °C, including CD14, CD90, CD166, CD144, CD73, CD105, CD45, CD31, CD29, and the HLA-DR isotype. Cell suspensions with isotype-matched immunoglobulins were used as controls. After three washes with PBS, the labeled samples were analyzed using a FACSAria II flow cytometer (BD Biosciences, San Jose, CA).

### In vitro assessment of osteogenic differentiation

Cells were cultured in osteo-inductive medium (alpha-Minimum Essential Medium [α-MEM] containing 0.1 μM dexamethasone, 10 mM β-sodium glycerophosphate, 50 μM ascorbic acid, and 10% FBS) for 21 days, with a half-volume change performed every 3 days. Von Kossa staining was performed to detect calcified matrix precipitation. Cells were fixed in neutral formaldehyde solution for 1 h. After washing with deionized water, 2% silver nitrate solution was added for a 10-min reaction at 37 °C in the dark, a 15-min exposure time, and washing in deionized water. The presence of calcified matrix precipitation was assessed using an inverted phase-contrast microscope. Cells in the control group were cultured in α-MEM plus 10% FBS for 3 weeks.

### In vitro assessment of adipogenic differentiation

Cells were cultured in adipo-inductive medium (α-MEM containing 1 μM dexamethasone, 200 μM indomethacin, 10 μM insulin, 0.5 mM isobutyl methylxanthine, and 10% FBS), with a half-volume change performed every 3 days. After 3 weeks, the cells were fixed with neutral formaldehyde for 10 min at room temperature; this was followed by staining with Oil Red O and counterstaining of cell nuclei with alum hematoxylin to assess the degree of adipogenesis. Fat droplets were observed using an inverted phase-contrast microscope. The control cells were MSCs cultured in the aforementioned cell expansion medium.

### Preparation of cytotoxic T lymphocytes (CTLs)

First, peripheral blood mononuclear cells (PBMCs) were isolated from heparinized venous blood samples collected from healthy adults using Ficoll Histopaque (Sigma Chemical Co., St. Louis, MO) density gradient centrifugation. Subsequently, an EasySep™ Human T Cell Isolation Kit (STEMCELL Technologies Inc.) was used to isolate T cells from the PBMCs. Viable human T cells were seeded in Roswell Park Memorial Institute (RPMI)-1640 complete medium at a density of 1 × 10^6^ cells/mL. To activate the T cells, 25 µL/mL of ImmunoCult™ Human CD3/CD28/CD2 T Cell Activator and 100 U/mL of hIL-2 were added to the cell suspension. The cells were subsequently incubated at 37 °C with 5% CO_2_ for up to 3 days. To induce T cell expansion after 3 days of activation, the T cells were cultured in RPMI-1640 complete medium containing 100 U/mL of hIL-2 and subcultured once every 2–3 days to maintain a cell density of 1 × 10^6^ cells/mL. After 12 days of culture, the CTLs were collected for subsequent experiments.

### In vitro assessment of immune cell-induced tumor cell destruction

Tumor cells were plated at a density of 2,000 or 4,000 cells/well in either 96-well flat-bottomed plates or the lower chamber of a transwell system (Corning, NY, USA) in DMEM supplemented with 10% FBS. MSCs were plated at a density of 2,000 or 40,000 cells/well in either 96-well flat-bottomed plates or the upper chamber of a transwell system (Corning, NY, USA) in DMEM supplemented with 10% FBS. The following day, natural killer (NK) or T cells were added to the cultures on top of the tumor cell layer at different effect-to-target ratios in each well in RPMI 1640 medium containing 10% FBS supplemented with hIL-2 to reach a final concentration of 100 IU/mL. Luciferase activity was measured after 24 h and 48 h.

### Tumor formation assay

All protocols involving animals were approved by the Animal Care and Use Committee of the Beijing Institute of Biotechnology and performed in accordance with the National Institutes of Health Guidelines for the Care and Use of Laboratory Animals. To assess the tumor formation capacity, 3 × 10^6^ MC38 murine colon adenocarcinoma cells, with or without 3 × 10^6^ hLC-MSCs, were suspended in a 100-μL volume at a 1:1 ratio in either standard RPMI 1640 medium alone or medium with Matrigel matrix (BD Biosciences), and transplanted subcutaneously into 4- to 5-week-old C57BL/6N mice. Tumor formation was monitored weekly.

### Statistical analysis

All data are presented as the mean with standard deviations (SD) unless stated otherwise. Statistical analyses were performed using SPSS software (version 22.0; IBM Corp., Armonk, NY). For all quantitative measurements, a normal distribution was assumed, and differences between two groups were determined using unpaired, two-tailed Student’s *t*-tests. Each measurement was based on at least three independent replicates.

## Results

### Isolation of hLC-MSCs

**Fig. 1:**
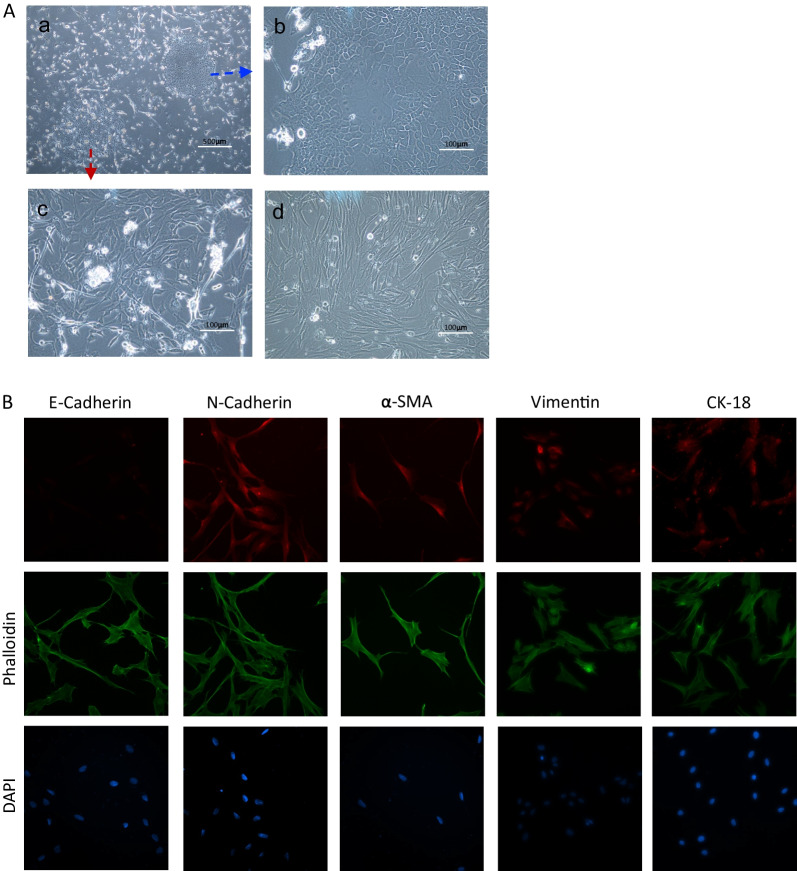
Morphology and immunophenotyping of hLC-MSCs. **A** (a) Representative images of primary cells arising from NSCLC tissues. Magnification, ×40. (b–d) The resultant distinct, purified colonies of epithelial tumors cells (b) or hLC-MSCs (c–d). Magnification ×200. **B** Representative images of hLC-MSC clones that were subjected to immunocytochemical staining. The hLC-MSCs were negative for E-cadherin and positive for N-cadherin, vimentin, α-SMA, and CK18 expression. Cytoskeletal proteins were stained using phalloidin, and cell nuclei were stained with DAPI. *hLC-MSCs* human lung cancer-derived mesenchymal stem cells; *NSCLC* non-small cell lung cancer; *α-SMA* alpha smooth muscle actin; *CK18* cytokeratin 18; *DAPI* 4’,6-diamidino-2-phenylindole

After 8 days of culture in conditions conducive to the growth of MSCs, the cells that remained adhered to the Petri dish formed several cell colonies (Fig. 1A-a). Cobblestone-like cell colonies, determined to be primary lung cancer cells, were observed (Fig. 1A-b), in addition to MSC-like cells that exhibited a long, spindle-shaped morphology and were abundant in all cultures (Fig. 1A-c). Single-cell suspensions were re-plated to increase the purity of the hLC-MSC clones, and several assays were conducted to confirm the low degree of contamination with other cell types, especially cancer and endothelial cells, to allow for cellular phenotyping (Fig. 1A-d). Immunophenotyping of the hLC-MSCs was performed based on the immunofluorescent labeling of various molecules, including E-cadherin (a marker of epithelial cells), N-cadherin and vimentin (markers of mesenchymal cells), α-smooth muscle actin (α-SMA) (a fibroblast marker), and cytokeratin 18 (CK18) (a malignant tumor marker). The hLC-MSCs isolated in the present study did not express E-cadherin, whereas they did express N-cadherin, vimentin, α-SMA, and CK18 (Fig. 1B).

### Surface markers expressed by hLC-MSCs

**Fig. 2:**
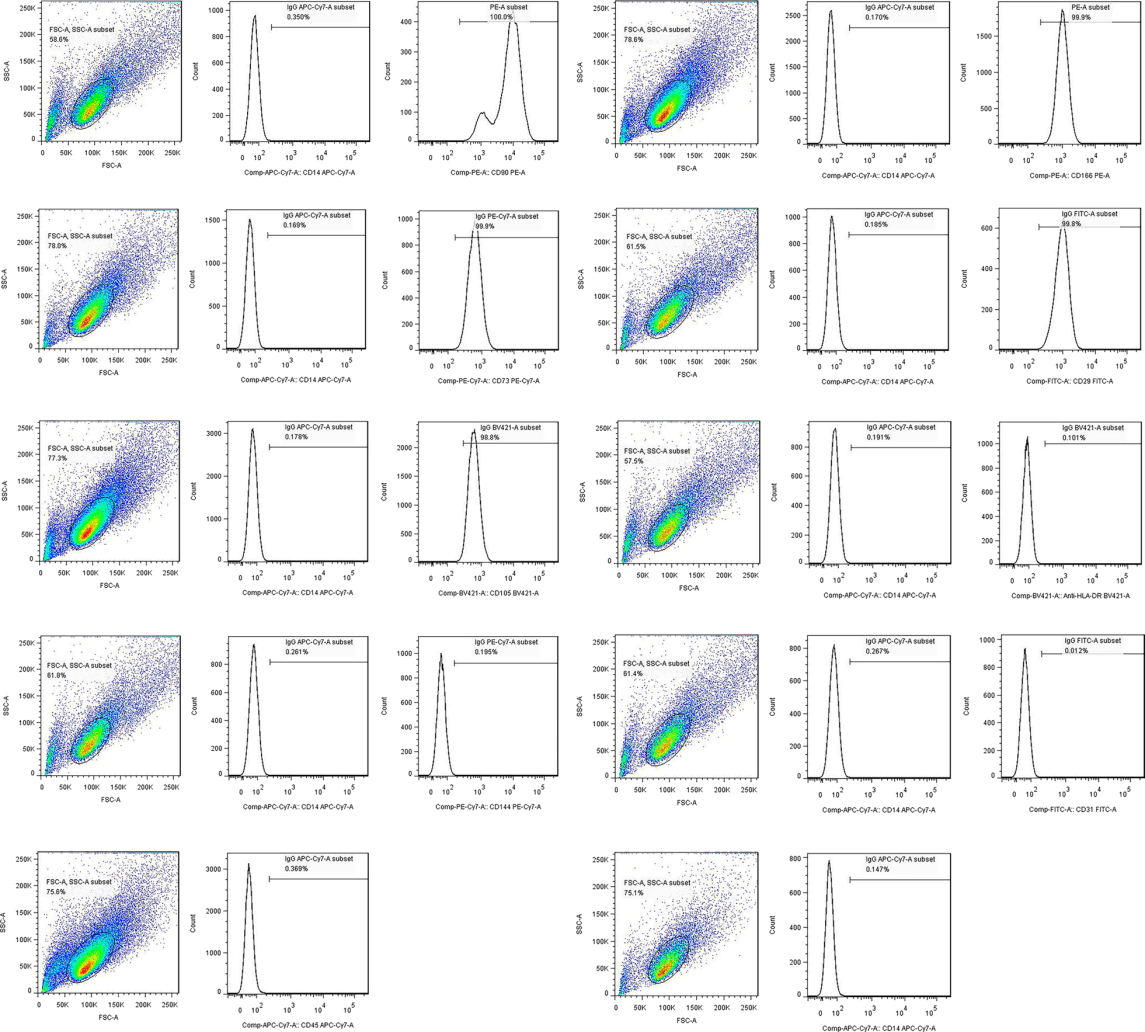
Surface antigens expressed by hLC-MSCs. Surface antigens expressed on hLC-MSCs were analyzed using flow cytometry. The hLC-MSCs were positive for CD90, CD166, CD73, CD29 and CD105, and negative for CD14, CD45, CD31, CD144, and HLA-DR. *hLC-MSCs* human lung cancer-derived mesenchymal stem cells; *CD* cluster of differentiation; *HLA-DR* human leukocyte antigen DR isotype

To determine whether the colony-forming cells (hLC-MSCs) expressed the same characteristic surface antigens as MSCs, flow cytometric analysis was performed, which confirmed that these cells did in fact express a set of MSC markers, including CD90, CD166, CD73, CD29, and CD105 (Fig. 2). Antibodies that target several of these antigens are routinely used to characterize expanded mesenchymal cell populations. In contrast, the cells did not express the lipopolysaccharide receptor CD14, the leukocyte common antigen CD45, the endothelial cell marker CD31, or the epithelial cell marker CD144. Moreover, the cells did not express HLA-DR. Collectively, these data confirmed that the isolated MSC-like cells exhibited surface marker expression patterns typically observed in MSCs, which was in accordance with the accepted phenotypic markers for hMSCs described previously [21].

### Multilineage differentiation capacity of hLC-MSCs

**Fig. 3:**
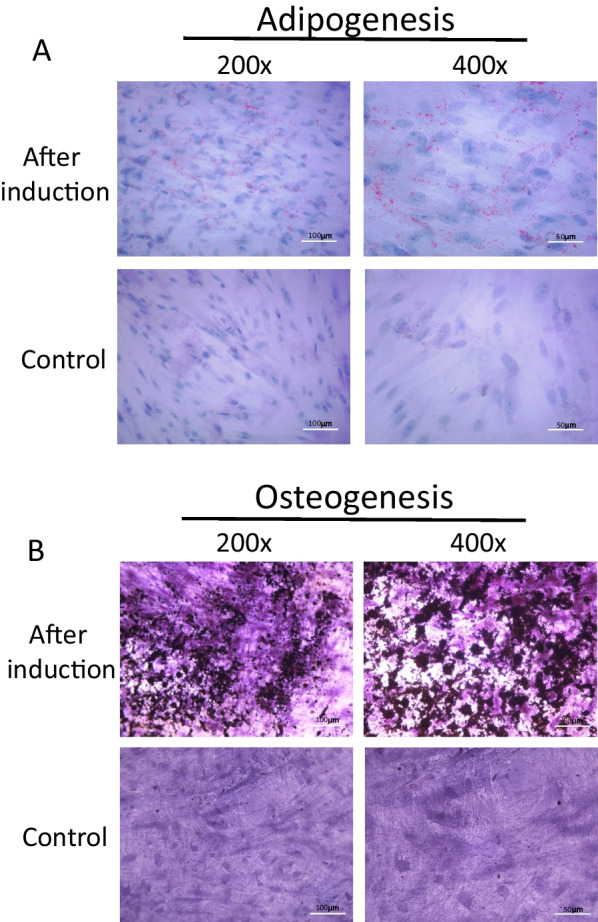
Differentiation potential of hLC-MSCs. **A** Representative images of hLC-MSCs that had differentiated into adipocytes, with positive Oil-Red-O staining of triglycerides. Most hLC-MSCs exhibited positive Oil-Red-O staining after being induced to differentiate into adipocytes for 21 days, whereas the control cells, which were not exposed to conditions conducive to adipocyte development, failed to undergo such differentiation (magnifications: ×200, ×400). **B** Representative images of hLC-MSCs that had differentiated into osteoblasts based on positive von Kossa staining of calcium deposition after three weeks of exposure to conditions specifically conducive to osteoblast development. The control cells failed to undergo such differentiation, as evidenced by the negative von Kossa staining (magnification: ×200, ×400). *hLC-MSCs* human lung cancer-derived mesenchymal stem cells

True MSCs have the capacity to undergo multipotent differentiation into cells of the adipogenic, osteogenic, and chondrogenic lineages when cultured in specific media [1, 22, 23]. Therefore, the cells were first cultured in a medium containing 1-methyl-3-isobutylxanthine, dexamethasone, insulin, and indomethacin to induce adipogenic differentiation [1]. After a two-week incubation, the hLC-MSCs had begun to undergo differentiation. In the third week, the hLC-MSCs had clearly differentiated into adipocytes (Fig. 3A), with lipid droplets with positive Oil Red O staining.

To promote osteogenic differentiation, the hLC-MSCs were cultured in a medium supplemented with dexamethasone, β-glycerophosphate, and ascorbate. Three weeks later, osteogenic differentiation products were detected using von Kossa staining, whereas the cells that had been cultured in normal media did not undergo such differentiation after culturing for 3–4 weeks or longer. Collectively, these data indicated that the isolated hLC-MSCs possessed the capacity to undergo multipotent differentiation under different culture conditions.

### hLC-MSCs enhance cancer cell growth in vivo

**Fig. 4:**
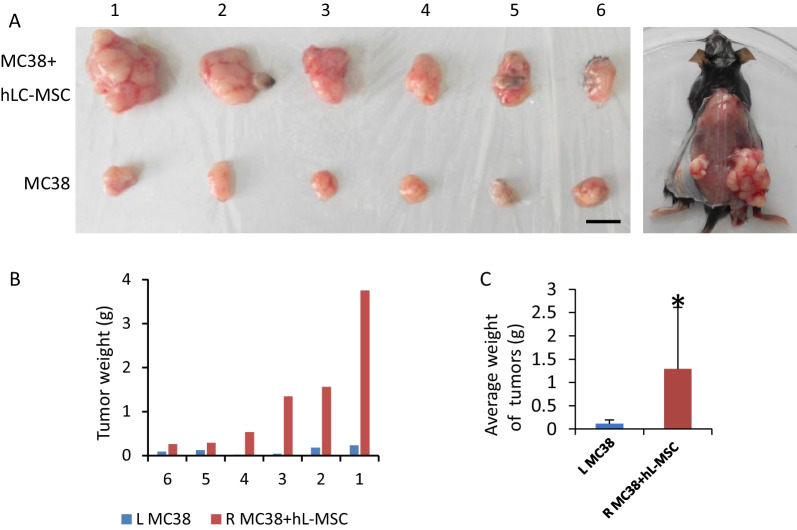
Human lung cancer-derived mesenchymal stem cells enhance tumor cell growth in vivo. **A** Representative images showing the formation of tumors in C57BL/N6 mice who were subcutaneously injected with either MC38 cells alone (left) or MC38 cells in combination with hLC-MSCs (right) (n = 6). **B**, **C** Tumor weights at the end of the experiment. The data are shown as the mean ± SD. *P < 0.05; **P < 0.01; ***P < 0.001. *MC38* murine colorectal adenocarcinoma cells; *hLC-MSC* human lung cancer-derived mesenchymal stem cells; *SD* standard deviation

Some studies have reported that MSCs promote tumor growth, whereas other have shown they suppress it. To evaluate the effect of hLC-MSCs on tumor formation in vivo, MC38 cells, a line of murine colorectal adenocarcinoma cells, were injected into the flanks of immune-competent C57BL/6N mice, either alone or in combination with hLC-MSCs. Tumors formed by the co-injection of MC38 cells and hLC-MSCs were significantly larger than those formed by the injection of MC38 cells alone. To examine the effect of hLC-MSCs on tumor growth in vivo, hLC-MSCs were also injected alone into C57BL/6N mice; this failed to induce tumor formation under the same conditions. After 6 weeks, the mice were euthanized and the tumors were dissected and weighed (Fig. 4A). The tumor weights in the MC38 + hLC-MSC group were significantly higher than those in the control group injected with tumor cells alone (Fig. 4B, C). These data demonstrate that the tumor-promoting abilities in mice with normal immunity were a specific property of the admixed MSCs or their derivatives.

### hLC-MSCs inhibit NK cell-mediated tumor destruction in contact and non-contact systems

**Fig. 5:**
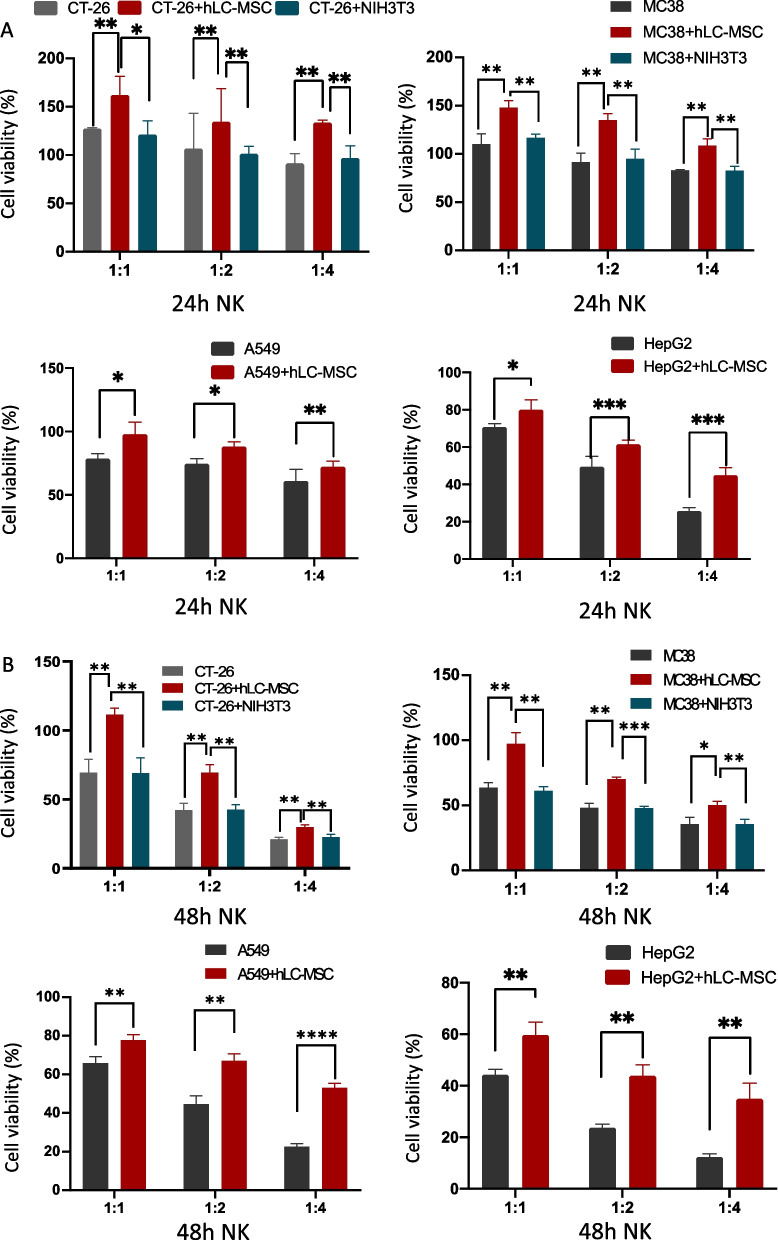
Human lung cancer-derived mesenchymal stem cells inhibit NK cell-mediated tumor destruction in vitro. Tumor cells were cultured in normal medium for 24 h (**A**) or 48 h (**B**) in the presence or absence of hLC-MSCs at hLC-MSC:NK cell ratios of 1:1, 1:2, and 1:4 in the contact system. The data are expressed as the mean ± SD based on data from three independent replicates. *Statistically significant (*P* < 0.05) difference compared with that of the cultures performed without hLC-MSCs. *NK* natural killer; *hLC-MSCs* human lung cancer-derived mesenchymal stem cells

**Fig. 6:**
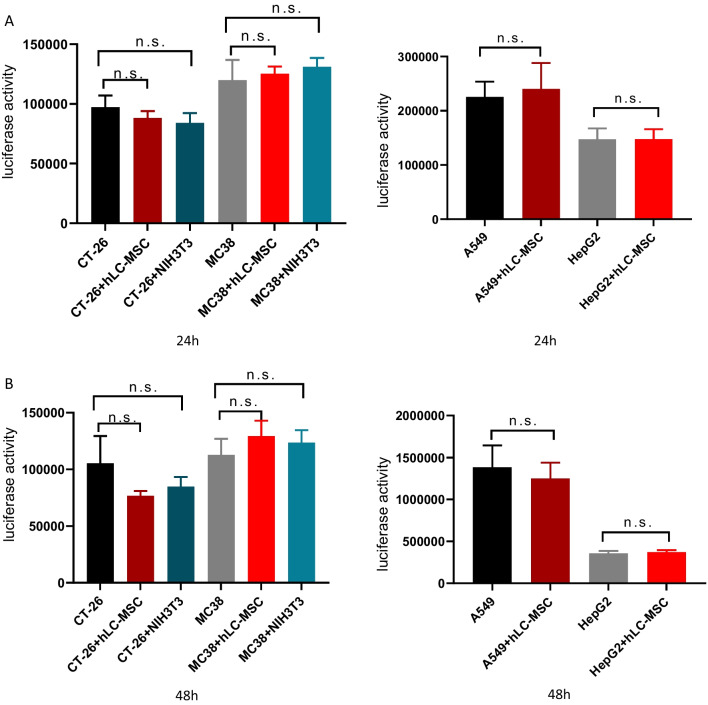
hLC-MSCs effects on tumor cell growth in an in vitro contact system. hLC-MSCs did not affect the kinetics of CT-26, MC-38, A549, or HepG2 tumor cells in vitro after 24 h (**A**) or 48 h (**B**) of exposure in the contact system. The histograms show the luciferase activity of the indicated cells cultured for 24 h or 48 h in vitro in the presence or absence of hLC-MSCs (n = 5). *P ≤ 0.05, **P ≤ 0.001, n.s. > 0.05 by Student’s *t*-test. The error bars represent the SD of three independent experiments. *hLC-MSC* human lung cancer-derived mesenchymal stem cells; *CT-26* an undifferentiated colon carcinoma cell line; *MC-38* a murine colon adenocarcinoma cell line; *A549* lung carcinoma epithelial cell line; *HepG2* a human liver cancer cell line; *n.s.* not significant; *SD* standard deviation

Tumor cells were co-cultured with hLC-MSCs (at a 1:1 ratio) or without hLC-MSCs at tumor cell:NK cell ratios of 1:0, 1:1, 1:2, 1:4, and 1:5 in a combined medium containing DMEM with 10% FBS plus MSC-conditioned medium. In the contact systems, as presented in Fig. 5A, B, hLC-MSCs inhibited the killing efficiency of NK cells in a dose-dependent manner; similar results were observed for the transwell system experiments, as presented in Fig. 8A, suggesting that soluble factors are involved in this process. To rule out the possibility that the effect of mass was mediating this outcome, the same experiments were repeated with NIH3T3 fibroblast or MRC-5 fetal lung fibroblast cell lines rather than hLC-MSCs at a ratio of 1:1. Little inhibitory effect was observed in either the contact cultures or the transwell systems (data not shown). To confirm that these effects were the result of the inhibition of NK cell-mediated destruction and not due to changes in cellular proliferation, luciferase values were compared with those of the tumor cells in the control group that had not been exposed to NK cells; no significant differences were observed between the tumor cell–MSC co-culture group, the tumor cell–control cell co-culture group, and the tumor cell only group (Figs. 6A, B and 8C). Collectively, these results suggest that the attenuation of the ability of NK cells to destroy tumor cells can be attributed to the hLC-MSC co-culture.

### hLC-MSCs inhibit T cell-mediated tumor destruction in contact and no-contact systems

**Fig. 7:**
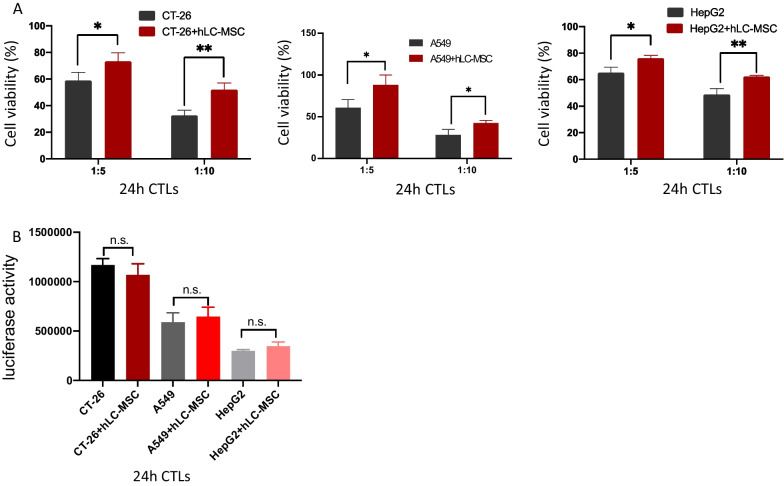
Human lung cancer-derived mesenchymal stem cells inhibit CTL-mediated tumor cell destruction in vitro. **A** Tumor cells were cultured in ordinary medium for 24 h in the presence or absence of hLC-MSCs, at hLC-MSC:CTLs ratios of 1:5 and 1:10 in a contact system. The in vitro cell viability ratios of the indicated cells are shown (n = 5). **B** hLC-MSCs did not affect the kinetics of CT-26, A549 or HepG2 tumor cells in vitro following 24 h of exposure in a contact system. The histograms show the luciferase activity of the indicated cells cultured in the presence or absence of hLC-MSCs (n = 5). The data are expressed as the mean ± SD from three independent replicates. *P ≤ 0.05, **P ≤ 0.001, n.s. > 0.05 by Student’s *t*-test. The error bars represent SD of three independent experiments. *CTL* cytotoxic T lymphocytes; *hLC-MSCs* human lung cancer-derived mesenchymal stem cells; *CT-26* an undifferentiated colon carcinoma cell line; *A549* lung carcinoma epithelial cell line; *HepG2* a human liver cancer cell line; *SD* standard deviation; *n.s.* not significant

**Fig. 8:**
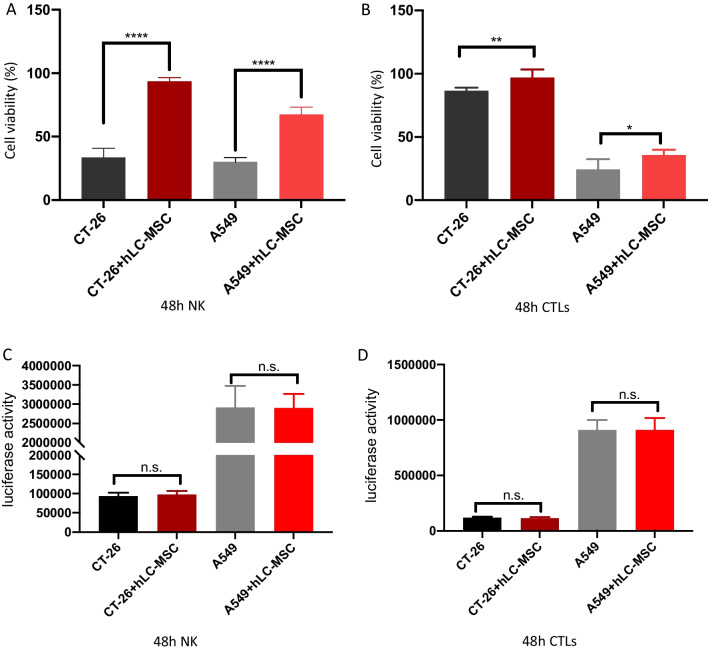
Human lung cancer-derived mesenchymal stem cells inhibit NK-/CTL-mediated tumor cell destructions in vitro. Tumor cells were cultured in ordinary medium for 48 h in the presence or absence of hLC-MSCs at hLC-MSC:CTL (**A**) or hLC-MSC:NK cell (**B**) ratios of 1:5 in a transwell system. The histograms show the viability of the indicated cells cultured for 48 h in vitro in the presence or absence of hLC-MSCs (n = 5). **C**, **D** hLC-MSCs did not affect the kinetics of the indicated cells when cultured in a transwell system containing tumor cells for 48 h. The histograms show the luciferase activity of the indicated cells cultured for 48 h in vitro in the presence or absence of hLC-MSCs (n = 5). *P ≤ 0.05, **P ≤ 0.001, n.s. > 0.05 by Student’s *t*-test. Error bars represent the SD of three independent experiments. *NK* natural killer; *CTL* cytotoxic T lymphocyte; *hLC-MSCs* human lung cancer-derived mesenchymal stem cells; *n.s.* not significant; *SD* standard deviation

Tumor cells were co-cultured with hLC-MSCs (at a 1:1 ratio) or without hLC-MSCs at tumor cell:T cell ratios of 1:0, 1:5, and 1:10 in a combined medium consisting of DMEM with 10% FBS plus MSC-conditioned medium. As presented in Fig. 7A, hLC-MSCs inhibited the killing efficiency of T cells in a dose-dependent manner. This inhibitory effect was conspicuous when MSCs and cancer cells were in physical contact, and this influence remained significant even when they were cultured separately (Fig. 8B). In addition, to rule out the possibility that the observed effect was related to mass in both the contact culture and transwell system, hLC-MSCs were replaced with NIH3T3 (or MRC-5) cells, which resulted in the absence of an inhibitory effect. The comparison of luciferase signals confirmed that the observed effects were not a result of changes in tumor cell proliferation (Figs. 7B, 8D). Collectively, these results suggest that the compromised T cell-mediated destruction of tumor cells can be attributed to the hLC-MSC co-culture.

## Discussion

MSCs have become a focal point of research in regenerative medicine and immunology because of their capacity for self-renewal, their ability to differentiate into multiple cell types, their tendency to be recruited to sites of inflammatory injury, and their immunosuppressive capabilities. During tumorigenesis, non-cancerous tissue-derived MSCs (such as bone marrow-derived MSCs [BM-MSCs]) are recruited to tumor sites where they become integrated into the tumor stroma where they are instructed to adapt to novel features and become tumor-resident MSCs. Therefore, the properties of tissue-resident MSCs are primarily determined by the tissue in which they reside and their physical location within those tissues [24].

Although numerous studies have investigated the correlation between noncancerous tissue-derived MSCs and tumor cells, transformed tumor-resident MSCs have not been adequately characterized to date in terms of their properties and their roles in modulating tumor growth and progression. The results of the present study indicate that MSCs are commonly present in the tumors of human patients with lung cancer, which is consistent with previous reports that MSCs are present in those with many other types of cancer [25, 26]. The study provides clear experimental evidence that indicates the isolated cells were, in fact, MSCs, and not cancer-associated fibroblasts. First, the hLC-MSCs demonstrated the capacity for multipotent differentiation by transforming into adipose and bone tissues when exposed to certain conditions in vitro. The hLC-MSCs also expressed cell surface markers that are commonly expressed on MSCs, such as CD29, CD73, CD90, CD166, and CD105, whereas hematopoietic markers such as CD14, CD31, and CD45 were absent. Additionally, unlike fibroblasts, these cells could be stably maintained in MSC-specific medium for several months without losing their capacity for multipotent differentiation.

Most previous studies that have conducted in vivo tumorigenic experiments in immunodeficient mice involved models or conditions that failed to objectively and accurately reflect the immunosuppressive effects of tumor-associated MSCs on cancer cell growth and proliferation. Thus, in the present study, C57BL/6N immunocompetent mice were used to more adequately reflect real-world conditions. The data strongly support the fact that MSCs exert tumor-promoting activity within the tumor microenvironment, an effect that appears to be at least partially due to their immunosuppressive capabilities. These results are consistent with those of previous studies; for example, in 2012, Ren et al. reported that murine lymphoma-derived MSCs (L-MSCs) exerted a much more pronounced effect on the promotion of tumor growth compared to that of matched BM-MSCs [27]. Tumor-associated MSCs differ from BM-MSCs in several ways; for instance, an in-depth in vivo analysis revealed that, unlike their BM-MSC counterparts, these L-MSCs produced high levels of the C–C motif chemokine receptor 2 (CCR2) ligands CCL2, CCL7, and CCL12, which promoted the recruitment of macrophages to tumor sites where they underwent a phenotypic shift to the tumor-promoting M2-like phenotype [27].

It has long been assumed that the primary mechanism through which the immune system achieves tumor cell destruction involves NK cells and major histocompatibility complex class I (MHC-I)-restricted CTLs [7]. Tumor cells that downregulate MHC-I molecules are protected from CTL-mediated destruction, although they are still susceptible to NK-mediated killing. Recently, human MSCs have been shown to exhibit immunosuppressive properties that affect NK and T-lymphocyte proliferation in an MHC-independent manner, bypassing the species barrier. MSCs may also be capable of inhibiting several functions of naïve and memory T cells, and they express negligible levels of MHC-II molecules, low levels of MHC-I molecules, and no co-stimulatory molecules [28–31]. Since MSCs themselves are not inherently immunogenic, they are incapable of eliciting allogeneic T cell responses [32]; this phenomenon has been reported to be mediated by the production of certain cytokines, such as transforming growth factor beta 1 (TGF-β1) and hepatocyte growth factor (HGF), rather than by the induction of apoptosis [17, 29, 33–35].

To test this assumption, several in vitro experiments were conducted. First, to test whether this effect was caused by direct contact or if it occurred via soluble mediators, tumor cells were co-cultured with hLC-MSCs in direct or transwell systems. Compared with the abundance seen in the blank control or the group involving mixed fibroblast populations, tumor cells in the hLC-MSC group were significantly more abundant, regardless of which co-culture system was used. To test whether this phenomenon was caused by tumor cell proliferation or the suppression of immune cell-mediated tumor destructions, the number of tumor cells was quantified in each group in which the immune cells were absent from the culture; those experiments revealed that the presence of hLC-MSCs had little impact on the proliferation of tumor cells among the different groups and confirmed that hLC-MSCs promoted tumor growth, at least in part, by inhibiting immune cell-mediated tumor cell destruction.

It is important to acknowledge that the present study was a preliminary exploration of the effects of MSCs on tumor cells, and further studies are required to investigate the underlying mechanisms. Both in vivo and in vitro studies have shown that murine BM-MSCs and human placental MSCs can induce tolerance in monocytes and a phenotypic shift in macrophages from an inflammatory phenotype to an immunosuppressive one that is characterized by increased IL-10 production and the expression of co-inhibitory molecules such as B7-H4 [36, 37]. Interleukin 6 (IL-6) is significantly enriched in the supernatant of cultured MSCs and exerts an immunosuppressive effect; however, other studies have shown that IL-6 can promote the expression of programmed death-ligand 1 to inhibit anti-tumor immunity [38, 39]. Collectively the findings of the present study and those previously conducted suggest that MSCs, tumor cells, and immune cells affect tumor growth and evolution through intricate regulatory mechanisms, which must be systematically investigated to gain a better understanding of the regulatory processes that control tumor growth and metastasis.

## Conclusions

The presence of mesenchymal stem cells has been confirmed in some solid tumors, where they serve as important components of the tumor microenvironment; however, their role in cancer has not been fully elucidated, and there have been contradictory findings reported in terms of whether they suppress or promote tumor growth or survival. This study investigated the functions of mesenchymal stem cells isolated from tumor tissues of a patient with non-small cell lung cancer. In vitro and in vivo experiments were performed to determine the characteristics of these isolated cells and their effects on immune cell-mediated destruction of tumor cells. The isolated human lung cancer-derived mesenchymal stem cells displayed the typical morphology and immunophenotype of MSCs, and the results confirmed that they were nontumorigenic and capable of undergoing multipotent differentiation. These isolated cells remarkably enhanced tumor growth when incorporated into systems alongside tumor cells in vivo. Importantly, in the presence of MSCs, the ability of peripheral blood mononuclear cell-derived natural killer cells and activated T cells to mediate tumor cell destruction was significantly compromised. These data support the notion that hLC-MSCs protect tumor cells from immune-mediated destruction by inhibiting the antitumor activities of NK and T cells, which could contribute to poorer outcomes. This study provides a good basis for further exploration of the mechanisms that regulate the interactions and effects between these cell types in various cancers.

## Data Availability

The datasets used and/or analyzed during the current study are available from the corresponding author on reasonable request.

## Abbreviations

α-MEM: Alpha-Minimum Essential Medium
α-SMA: Alpha-smooth muscle actin
BM-MSCs: Bone marrow-derived mesenchymal stem cells
CCL: Chemokine ligand
CCR2: C-C motif chemokine receptor 2
CD: Cluster of differentiation
CK18: Cytokeratin 18
CTLs: Cytotoxic T lymphocytes
FBS: Fetal bovine serum
DAPI: 4’,6-Diamidino-2-phenylindole
hIL-2: Human interleukin 2
HGF: Hepatocyte growth factor
HLA: Human leukocyte antigen
hLC-MSC: Human lung cancer-derived mesenchymal stem cells
MHC-1: Major histocompatibility complex class 1
L-MSCs: Lymphoma-derived mesenchymal stem cells
MSC: Mesenchymal stem cells
NK: Natural killer
PBMCs: Peripheral blood mononuclear cells
PBS: Phosphate-buffered saline
RPMI: Roswell Park Memorial Institute
SD: Standard deviation
SEM: Standard error of the mean
TGF-β1: Transforming growth factor beta one
VSV-G: Vesicular stomatitis virus G
